# Antigenic Characterization of Recombinant Hemagglutinin Proteins Derived from Different Avian Influenza Virus Subtypes

**DOI:** 10.1371/journal.pone.0009097

**Published:** 2010-02-05

**Authors:** Matthias Mueller, Sandra Renzullo, Roxann Brooks, Nicolas Ruggli, Martin A. Hofmann

**Affiliations:** Institute of Virology and Immunoprophylaxis (IVI), Mittelhaeusern, Switzerland; Hong Kong University, Hong Kong

## Abstract

Since the advent of highly pathogenic variants of avian influenza virus (HPAIV), the main focus of avian influenza research has been the characterization and detection of HPAIV hemagglutinin (HA) from H5 and H7 subtypes. However, due to the high mutation and reassortation rate of influenza viruses, in theory any influenza strain may acquire increased pathogenicity irrespective of its subtype. A comprehensive antigenic characterization of influenza viruses encompassing all 16 HA and 9 neuraminidase subtypes will provide information useful for the design of differential diagnostic tools, and possibly, vaccines. We have expressed recombinant HA proteins from 3 different influenza virus HA subtypes in the baculovirus system. These proteins were used to generate polyclonal rabbit antisera, which were subsequently employed in epitope scanning analysis using peptide libraries spanning the entire HA. Here, we report the identification and characterization of linear, HA subtype-specific as well as inter subtype-conserved epitopes along the HA proteins. Selected subtype-specific epitopes were shown to be suitable for the differentiation of anti-HA antibodies in an ELISA.

## Introduction

Influenza viruses (IV) belong to the family of *Orthomyxoviridae*, which are characterized by a segmented and single-stranded negative sense RNA genome. 8 segments encode 11 viral proteins, the internal proteins (nucleoprotein, NP; matrix proteins, M1 and M2; nonstructural proteins, NS1 and NS2; polymerase basic proteins, PB1, PB1-F2 and PB2; polymerase acidic protein, PA) and the 2 surface proteins (hemagglutinin, HA and neuraminidase, NA). While the internal proteins are well conserved among all IV strains, HA exists in 16 and NA in 9 different subtypes, respectively. Hence, IV subtype classification is based on the HA-NA combination. Wild aquatic birds are the natural reservoir of all HA and NA subtypes of avian IV (AIV), however, the theoretical subtype spectrum is reduced by a preference of each HA to be associated with a certain subset of NA subtypes [1]. Several subtypes are able to infect also mammalian hosts, e.g. humans (H1N1, H2N1, H3N2), horses (H7N7, H3N8) and pigs (H1N1, H1N2, H3N2, H4N6) [2]. In wild aquatic birds, low pathogenic AIV (LPAIV) replicates usually asymptomatically in the intestinal tract. However, mutations in the viral genome of H5 and H7 subtypes, specifically in the HA gene, can lead to the emergence of highly pathogenic AIV (HPAIV) upon transmission to susceptible gallinaceous poultry [3]. HPAIV replicate in all tissues causing peracute to acute fatal disease in poultry and mild to severe disease in wild birds, depending on the virus strain and bird species, age and condition [4]. Human influenza strains in general cause seasonal flu characterized by respiratory symptoms and, if accompanied by a secondary, mostly bacterial infection, these IV can lead to fatal cases in young, immunocompromized or elderly patients. However, the high mutation rate (antigenic drift) of IV and random reassortation of genomic segments (antigenic shift) in animals simultaneously infected with different IV subtypes may lead to an adaptation of HPAIV to the human host, generating a new highly virulent pandemic strain.

The major factor for IV infectivity is the HA surface protein that mediates binding of the virus to the host-specific cell surface receptors α2,3-sialic acid (SA) and α2,6-SA in birds and mammals, respectively [2]. Thus, HA is the prime target for the development of new diagnostic, therapeutic and preventive tools, and therefore a comprehensive antigenic characterization of the IV HA is needed. In order to rapidly recognize outbreaks of new IV strains with increased pathogenicity, efficient surveillance is necessary, which is able to detect all IV subtypes, not only the epidemically most relevant ones.

Traditional identification and subtyping of IV prescribed by the Office International des Epizooties (OIE) is based on virus isolation followed by serological tests, namely agar gel immunodiffusion (AGID) to identify any IV, and hemagglutination inhibition (HI) [5], using HA and NA subtype-specific reference sera, for subtyping. These traditional methods have been mostly replaced by molecular methods in recent years, i.e. using RT-PCR and nucleotide sequencing of the HA and NA genes. Detection and subtyping of IV antibodies on the other hand still mostly relies on HI using (inactivated) IV reference strains representing the entire repertoire of HA and NA subtypes. ELISA-based antibody subtyping has only been described very recently. However, these ELISA employ only 1 individual antigen [6], [7], [8] and are, tentatively, monospecific for a selected HA or NA subtype. Novel technologies such as multiplex fluorescent microsphere immunoassays (FMIA) employing more than 1 IV antigen [9] show, that antibody differentiating immunoassays are possible. Until now no multiplex approach is available for the subtyping of IV antibodies based on HA and NA serotype differentiation.

Initial antigenic characterization of IV HA protein for basic understanding of serological assays has revealed the importance of epitope conformation at the receptor-binding site formed by HA monomer folding and trimerisation [10]. The use of correctly folded HA was considered as a prerequisite for the detection and characterization of neutralizing epitopes, which are the mayor target of the virus neutralizing humoral immune response. This was supported by the observation that synthetic HA peptides failed to function as epitopes recognized by neutralizing monoclonal antibodies [11], [12], [13], [14]. However, recent findings that neutralizing antibodies are produced upon experimental vaccination with a bacterially expressed, detergent-treated and unfolded HA have shown that a virus-neutralizing immune response is possible independent of conformational epitope formation [15], [16]. Recent studies demonstrated that antibodies binding to linear HA epitopes achieve neutralizing activity by inhibiting the pH-dependent HA conformational rearrangement [17], [18], [19]. These findings show the existence of linear epitopes on IV HA and support their role during humoral immune response. The question is raised, if such epitopes represent a new repertoire of tools for serologic surveillance assays and if they are valid targets for serologic IV subtype differentiation. If they are present in a sufficient number, they may stimulate antibody production high enough to be detectable independent of the property to be neutralizing or not. Thus, linear epitopes present on HA should be included in the antigenic characterization of IV.

With the antigenic analysis of all HA and NA subtypes, a comprehensive picture of AIV HA antigenicity can be established which can be used to design highly specific differentiation tools.

Several studies have provided evidence that both HA subtype-specific as well as inter subtype-conserved epitopes do exist [20], [21], [22], [23], [24]. The primary aim of the present study was to determine, whether AIV subtype-specific linear epitopes on the HA protein can be detected with sera from animals immunized with recombinant AIV HA of the homologous subtype. We show that numerous linear, both inter subtype-conserved as well as subtype-specific epitopes exist on HA proteins derived from different AIV HA subtypes, based on differences in the reactivity patterns of homologous and heterologous antisera. Epitope mapping was performed by peptide scanning using libraries of overlapping peptides representing the entire HA from 3 different HA subtypes (H4, H5, H12), and rabbit antisera raised against the corresponding recombinant HA proteins. This approach allowed to elucidate the repertoire of subtype-specific and inter subtype-conserved epitopes among the studied AIV surface proteins. Our data provide evidence that all 3 HA subtypes analyzed carry linear epitopes in both the HA1 and HA2 ectodomains. In addition, synthetic peptides representing such epitopes were shown to be suitable as subtype-specific antigens for differential ELISA development.

## Results

**Figure 1:**
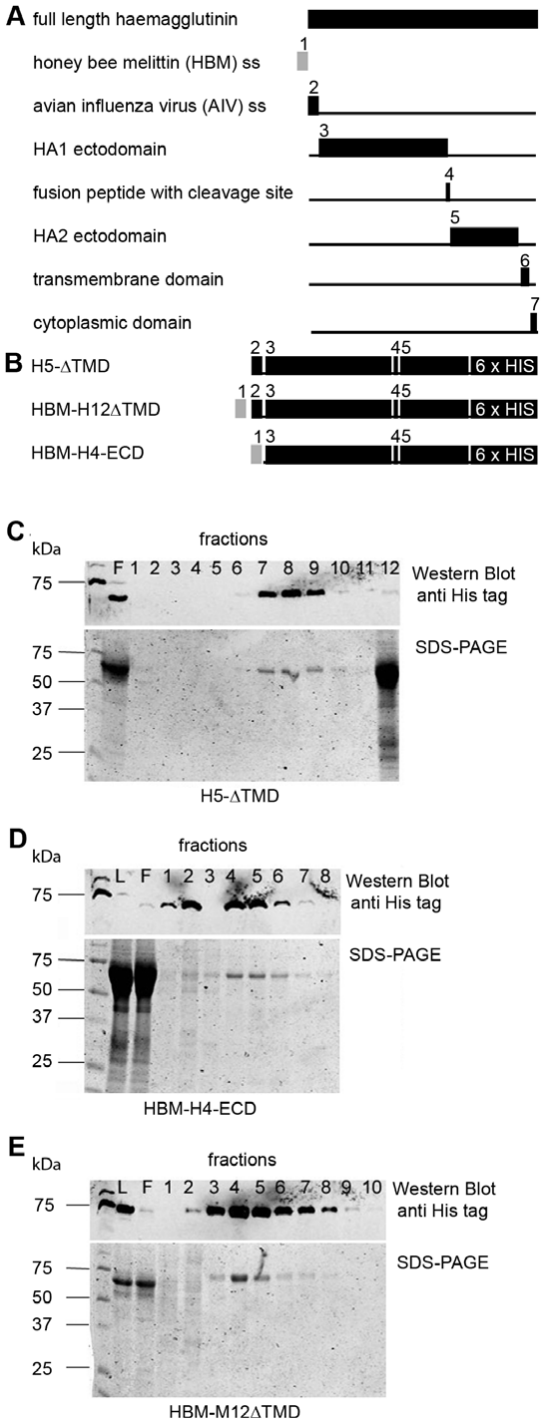
Construction and purification of recombinant AIV HA. Schematic drawing of the full-length HA protein and the relative location of the domains (black boxes) used for recombinant protein expression. The honeybee melittin (HBM) secretion signal (SS) is shown in grey. (B) Schematic illustration of recombinant HA proteins, shown as fusion of subtype-specific HA domains with the AIV ss or the HBM ss and a C-terminal 6xHis tag. (C-E) Western blot and SDS-PAGE analyses of cell culture supernatant (ccs) after Ni-NTA affinity chromatography. All proteins were secreted in the ccs in an uncleaved form and purified following an identical purification procedure. Differences in the number of positive elution fractions in the Western blot result from different quantities of recombinant protein bound to the Ni-NTA-column. SDS-PAGE results indicated that the most concentrated elution fractions contained almost exclusively the purified HA. Purification fractions are indicated with numbers, F =  flow through fraction, L =  load fraction.

**Figure 2:**
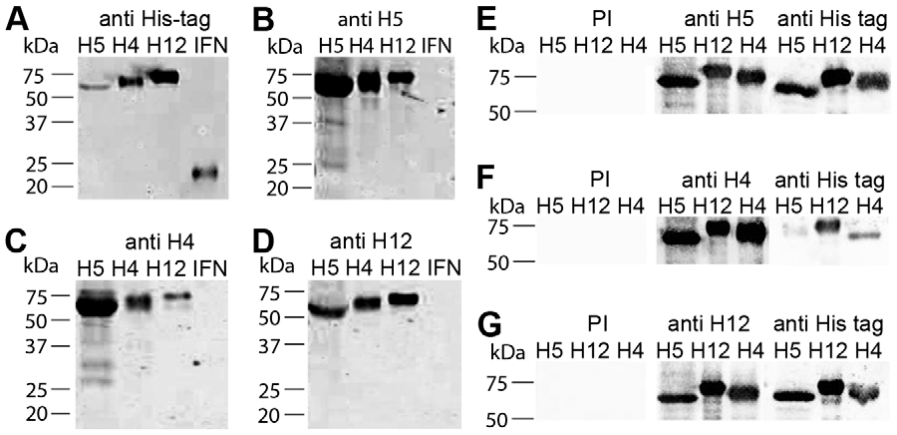
Western blot analyses with pre-immune sera and antisera derived from immunized rabbits. Unpurified recombinant H5 HA, H4 HA and H12 HA and a C-terminal 6x His tagged porcine IFN were blotted onto nitrocellulose membranes and analyzed with a monoclonal antibody against the His tag (A) or with serum from the 2nd bleeding (56 days post immunisation) from rabbits, immunized either with purified recombinant HA H5 (B), H4 (C) or H12 (D), respectively. Purified recombinant H5, H12 and H4 HA were blotted onto nitrocellulose membranes and analyzed with pre-immune rabbit sera (PI), a monoclonal antibody against the His tag and serum from the 3rd bleeding (114 days post infection) from rabbits, immunized with purified recombinant HA H5 (E), H4 (F) or H12 (G).

To test the effect of recombinant protein conformation on expression and secretion efficiency, recombinant H5, H4 and H12 HA was expressed via recombinant baculoviruses either as full length protein or as transmembrane domain deleted (ΔTMD) peptide containing the HA1 and HA2 ectodomains (Fig. 1A and B). No significant difference in secretion efficiency was observed using either the authentic AIV or the HBM secretion signal. Recombinant proteins lacking the TMD were efficiently expressed and secreted into the cell culture supernatant, and could be used as starting material for His tag affinity purification. Buffer exchange from cell medium to carbonate buffer and purification using HisTrap-Ni-NTA columns with an imidazole gradient resulted in highly pure and concentrated HA protein fractions, yielding up to 1 mg recombinant protein per 50 ml expression culture (Fig. 1C-E). This preparations were used to immunize rabbits. Pre-immune sera of all rabbits did not recognise the recombinant HA proteins whereas sera after immunization were highly reactive with all 3 recombinant HA, both before (Fig. 2A-D) and after affinity purification (Fig. 2E-G) of the HA proteins.

### Epitope mapping: Homologous system

**Figure 3:**
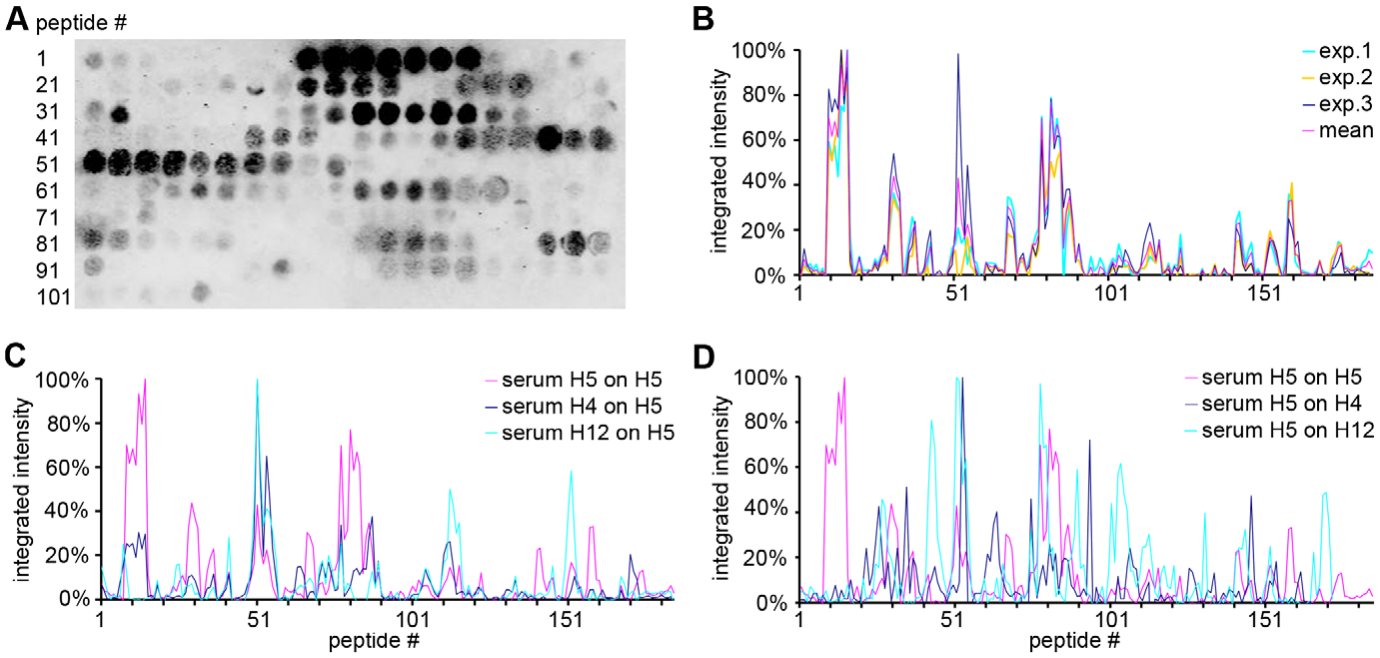
Visualization of data obtained from the peptide scanning analyses. The signal intensity of each peptide on the dot blot membrane is shown as integrated intensity after subtraction of unspecific background and secondary antibody-related signals. Relative reactivity values are normalized by setting the highest value of each experiment at 100%. Sera were tested on membranes containing their homologous antigen. Examples of H5 HA dot blot and homologous reactivity patterns obtained by 3-fold probing and stripping of the membrane are shown in A and B, respectively. Cross-reactivity of sera was identified by testing each serum with each heterologous membrane; a representative plot obtained with sera against H5, H4 and H12 on the membrane containing H5-specific peptides is shown in C. Furthermore, cross-reactive epitopes were also identified by testing each of the sera on their respective heterologous membranes, as shown for serum against H5 on membranes representing H4 or H12 (D).

To identify peptides that represent epitopes recognised by polyclonal antibodies, and to differentiate between subtype-specific and inter subtype-conserved epitopes, peptide scanning was performed with 15 amino acid (aa) long peptides overlapping by 12 aa and representing the complete set of linear epitopes of each expressed HA on a nitrocellulose dot blot membrane. Unspecific signals resulting from direct binding of the secondary antibody to the peptides were subtracted from the raw signal in every experiment to obtain sera-specific net reactivities. These signal intensities, shown in arbitrary units, were normalized to the highest value set as 100%. The normalized intensities of all peptides representing 1 HA protein were plotted against the peptide numbers along the HA protein in a diagram to show antigenicity curves for each of the recombinant H5, H4 and H12 HA. H5 contained the fewest reactive peptides and H4 the most. However, the integrated intensities were higher for H5 than for H4 and H12. Furthermore, epitopes present on H5 HA were found to be more concentrated to separated epitope-containing areas (Fig. 3A and B) whereas epitopes on the H4 and the H12 HA were found to be more evenly distributed along the entire protein (data not shown).

**Figure 4:**
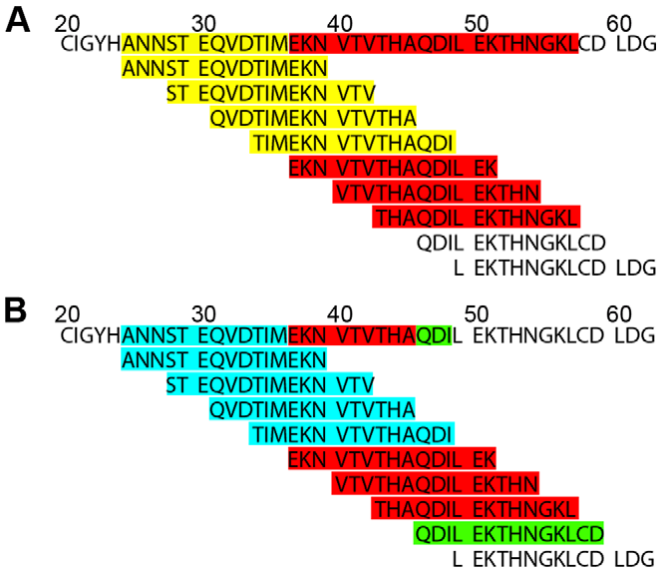
Principle of generation of semi-quantitative antigenic maps. Integrated intensities from peptide scanning analyses were transferred to the aa sequence as shown here for the HA aa sequence from HPAIV A/tufted duck/Switzerland/V504/06(H5N1) within the sequence range from aa position 20 to 63, as indicated. (A) Semi quantitative display of the gross signal intensities in color-coded categories: 100%–40% (red), 40%–30% (yellow). (B) Semi-quantitative display of the net signal intensities in color code categories: 100%–70% (red), 70%–40% (green) and 40%–20% (light blue).

**Figure 5:**
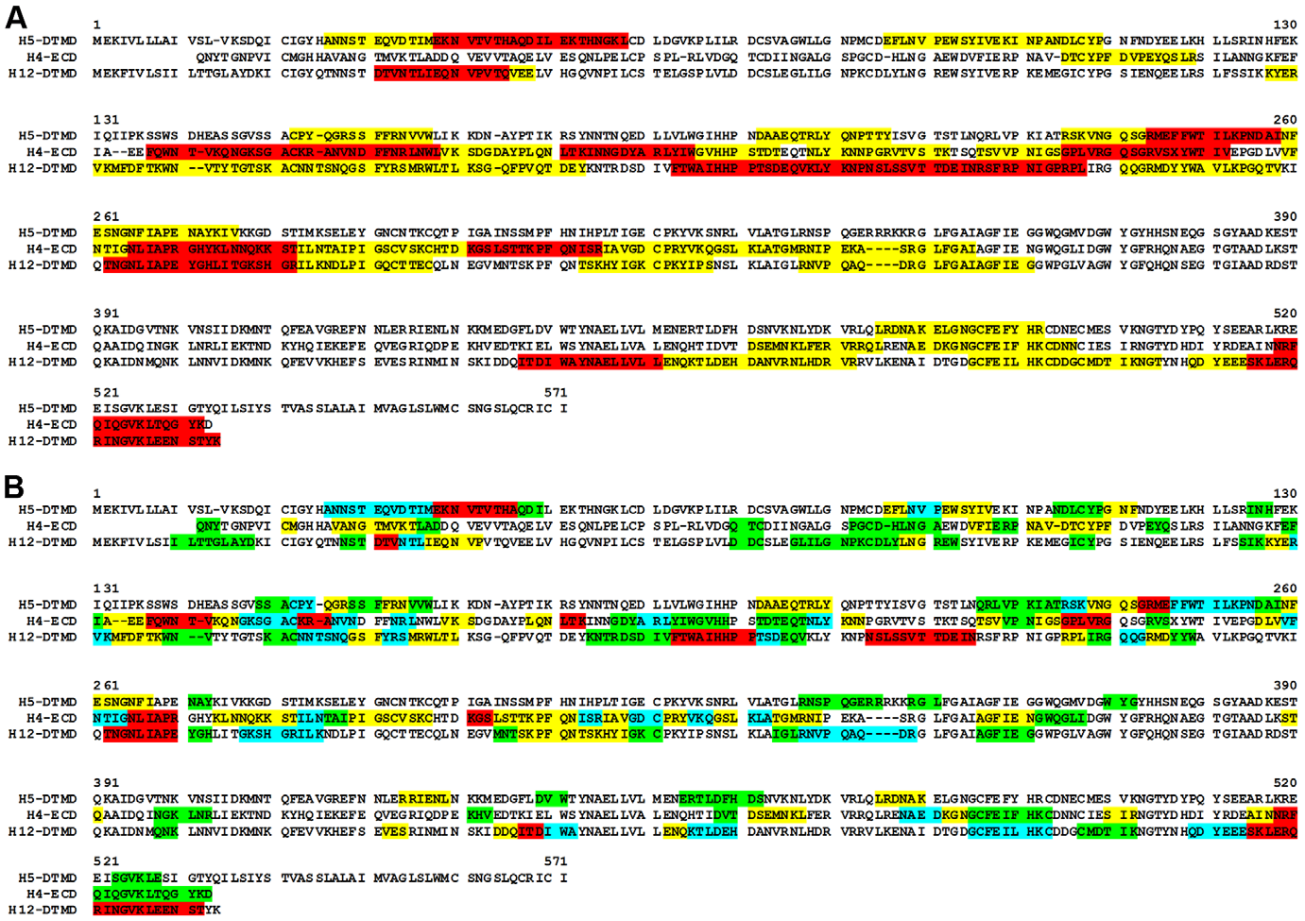
Antigenic maps of AIV HA domains reacting with their homologous sera. Color code indicates (A) the gross signal intensities found (compare to Fig. 4A, 100%–40% (red) and 40%–30% (yellow)) and (B) the net signal intensities found (compare to Fig. 4B, 100%–70% (red), 70%–40% (yellow) and 40%–20% (light blue)).

To generate an antigenic map based on the protein primary structure, the signal intensities from the antigenicity plots were superimposed onto the aa sequences of the expressed proteins by performing a semi quantitative and color-coded visualization of reactive peptides. Interestingly, some peptides reacted strongly whereas the next, overlapping peptide reacted only weakly or not at all with the rabbit sera. Epitope scanning was analyzed first by considering the complete length of strongly reacting peptides (Fig. 4A). Such an analysis allows generating a map of the localisation and distribution of epitope-containing regions (Fig. 5A). An alignment of the aa sequences of all 3 antigens used indicates that antibodies presented in the homologous antisera recognize the majority of peptides of H4 and H12 along the HA_1_ chain (aa 130 to 350), whereas in H5, the binding sites were allocated in clearly separated regions located within the HA_1_. The HA_2_ ectodomain shared only 1 antibody binding region in all 3 antigens.

To increase the resolution of antibody binding sites in all reactive peptides, weakly or non-reacting peptides were subtracted from neighbouring highly reactive ones (Fig. 4B). The peptides were color-coded according to their normalized dot blot intensities in steps of 100% to 70%, 70% to 40%, 40% to 20% and 20% to 10% integrated intensity, respectively. Furthermore, regions with overlapping peptides of different signal intensities were color-coded according to the less reactive peptide (net signal intensity) in order to identify antibody binding sites within the epitopes [25]. This analysis also demonstrated that the reactivity of antibody binding sites differs within conserved antibody binding regions in the HA H4, H5 and H12 (Fig. 5B).

### Epitope mapping: Heterologous system and subtype-specific epitopes

**Figure 6:**
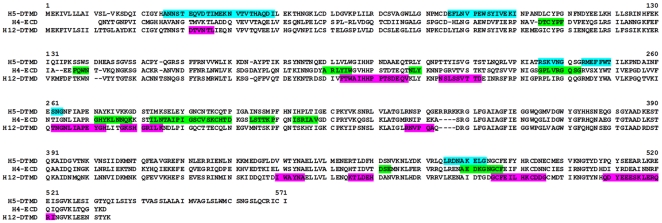
Subtype-specific epitopes. Integrated intensities from peptide scanning were transferred to the aa sequence of each AIV HA after subtraction of the heterologous from the homologous sera signals (H5, light blue; H4, green; H12, pink). All remaining epitopes are shown independent of their signal intensity in the homologous system.

In Western blot analyses, all 3 sera recognized all 3 antigens. In a second analysis the H5, H4 and H12 HA membranes were scanned for subtype-specific and cross-reactive linear epitopes in 2 different approaches. First, all membranes were tested individually with all sera to visualize cross-reactive epitopes, as shown for the H5 antigen (Fig. 3C). Additionally, sera against 1 of the 3 antigens were tested on membranes representing the 2 other antigens, to visualize the cross reactivity of the sera as shown for the serum anti H5 HA (Fig. 3D). The integrated membrane signals were plotted as for the homologous sera. Subtype-specific epitopes were obtained by subtracting the heterologous from the homologous signals. The reduced number of reactive peptides in H5 HA and the increased number of reactive peptides in H4 HA and H12 HA found previously in the homologous mapping correlated with fewer epitopes remaining on H5 compared to H4 and H12 HA after subtraction of the heterologous from the homologous sera signals (Fig. 6). When the epitope reactivities were compared, as shown in figure 5B and figure 6, in all 3 antigens the subtype-specific epitopes reacted moderately to strongly in the homologous systems (see also suppl. Table S1).

### ELISA

**Figure 7:**
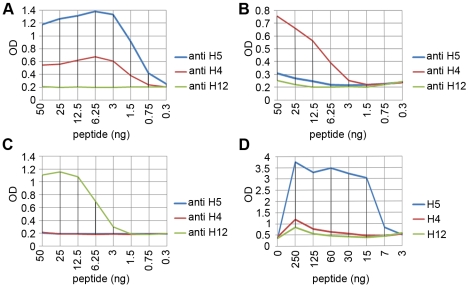
Antibody differentiation with polyclonal animal sera against different AIV HA subtypes tested in ELISA. (A) HA subtype-specific biotinylated peptide antigens (H5: biotin-Ttds-ANNSTEQVDTIMEKNVTVTHAQD-OH; H4: biotin-Ttds-DSEMNKLFERVRRQLRENAED KGNGCF-OH and for H12: biotin-Ttds-FTWAIHHPPTSDEQV-OH) were coated on ELISA plates at indicated amounts and tested with the 3 rabbit antisera (diluted 1∶500); H5 antigen (A), H4 (B), and H12 (C). Antigens H4, H5 and H12 HA were also tested with a chicken serum against H5 HA (diluted 1∶10) (D).

Based on the epitope mapping results the following peptides were selected as HA subtype-specific antigens in ELISA: H5: biotin-Ttds-ANNSTEQVDTIMEKNVTVTHAQD-OH; H4: biotin-Ttds-DSEMNKLFERVRRQLRENAEDKGNGCF-OH and for H12: biotin-Ttds- FTWAIHHPPTSDEQV-OH. As shown in Fig. 7, antibody subtype differentiation was possible both with the polyclonal rabbit sera (Fig. 7A-C) and with serum derived from a chicken that had been vaccinated against H5N9 (Fig 7D) in the indirect ELISA format, whereas in the blocking ELISA format no differentiation was possible (data not shown).

## Discussion

The HA of the 3 AIV HA subtypes H4, H5, and H12 were expressed as soluble recombinant 6xHis-tagged fusion protein in the baculovirus system. These 3 subtypes were chosen not primarily based on their epidemiological relevance, but because their HA genes are genetically quite different among each other, making it more likely that the recombinant HA proteins exhibit numerous differences in their epitopes. Immunization of rabbits with Ni-NTA affinity-purified recombinant HA resulted in the production of highly reactive antisera. However, these polyclonal sera could not be used for subtype differentiation using full-length antigens, because many of the epitopes presented on the antigens reacted at least weakly with the heterologous sera (Fig. 2B-G, Fig. 3C and D). Remarkably, the reactivity of the sera in Western blot analysis was even stronger with the heterologous antigens compared to their respective homologous antigens. This could be due to different antibody titers in the sera used or to different total antigen avidities caused by different numbers of homo- and heterologously reactive epitopes accessible on antigens transferred onto the nitrocellulose membrane. Nevertheless, the recognition of AIV HA subtype-specific and intra subtype-conserved epitopes in the form of overlapping linear synthetic peptides on PepSpot membranes with these sera was possible.

Since HA undergoes conformational changes mediated by an endosomal pH shift during infection, antibodies to conformational sites can be classified in 3 categories depending on their ability to recognize only the neutral form, only the acidified form, or both. Thus, the repertoire of linear epitopes in the analyzed HA proteins and of antibodies in the rabbit sera against the recombinant HA proteins found in this study might be similar to the repertoire of linear epitopes in and antibodies against native AIV.

Neutralizing epitopes in the HA1 receptor binding site are not conserved between subtypes [24]. Our results indicate that HA1 contains subtype-specific linear epitopes as well with a subtype-dependent variability in number and localisation among H4, H5 and H12 HA. The HA2 peptides of H4, H5 and H12 HA revealed only few subtype-specific epitopes (1 in H5 HA, 2 in H4 HA and 4 in H12 HA, respectively). This finding supports the model, that the fusion peptide-containing HA2 is less variable and harbours the majority of inter subtype-conserved epitopes [24].

Our results provide strong evidence, that several linear epitopes exist in AIV HA some of which are subtype-specific, whereas others are conserved among several subtypes. The location of the antibody-binding peptides, but not their sequence, was found to be conserved in all 3 antigens, suggesting that these peptides represent protein regions on the surface of the folded protein accessible to antibodies. The antibody binding sites differ within these conserved antibody binding regions in the HA H4, H5 and H12 (Fig. 5B). The lower number of epitopes found in H5 compared to H4 and H12 might explain the observation that in a mammalian system H5 HA is a less immunogenic antigen than HA from other subtypes [26]. Interestingly, this effect seems to be less pronounced in poultry vaccines [27]. The reasons for this are unknown. Upon analysis of the overall antigenic pattern recognized by hyperimmune rabbit sera compared to that recognized by hyperimmune chicken sera, no significant differences were observed (data not shown). This indicates that the species dependent immunogenicity of H5 HA cannot be explained by differences in the antigenic recognition of the aa primary structure. One explanation could be that the traditional formulation of inactivated whole-virus H5N1 vaccines [26], [27] contains additional epitopes of other viral proteins that may stimulate the immune response in a host species-dependent manner.

The peptide scanning approach provided informations which could be useful for the development of subtype-specific antibody differentiation tests. The antigenicity curves generated (Fig. 3C and D) show peptides from H5 that react specifically with antisera specific to H5. Such peptides can be assessed for their suitability for subtype-specific antibody differentiation. Additionally, the curves also show peptides from H5 HA with high reactivity to antisera specific to H4, H5 and H12 HA. These peptides mediate serologic cross-reactivity among different subtypes, hence they may be interesting candidates for the development of tailored multi HA-specific vaccines based on a combination of suitable recombinant peptide antigens. However, the analysis of the immunogenicity of these peptides was beyond the scope of this study.

Some of the peptides that were recognised in the peptide scanning analysis contain N-glycosylation sites. Since the insect cell expression system is known to be suitable for eukaryotic posttranslational glycan modifications, it is likely that the antigens used for immunization were glycosylated, even if insect cell glycosylation is not authentic to but a rudimentary form of mammalian glycosylation [28]. However, the fact that unglycosylated synthetic linear peptides are recognised by the sera raised against the recombinant HA proteins indicates, that epitope glycosylation is not a prerequisite for the detection by polyclonal antibodies; hence, epitope antigenicity is likely to be dependent on the protein primary structure, independent of their glycosylation state.

The development of new technologies for the generation of monoclonal antibodies, the expression of native recombinant antigens as well as improved analyses of protein structure and modification led to many detailed and important insights into the correlation of IV HA structure and antigenicity. However, after initial studies carried out in the 1980s the concept of linear epitopes to be used for AIV diagnostics and control by vaccination has not been further examined. Our data clearly show that such epitopes exist and could be further evaluated for their suitability as diagnostic or vaccine tools. During humoral immune response, viral antigens are presented to B cells either as intact or as recycled antigens, depending on the type of antigen presenting cell involved (for a comprehensive review see [29]). Viral antigen can be recycled and presented as short peptides that differ in their conformation from the native antigen. This might explain the selection of B cell clones that express antibodies reacting independent of the native antigen conformation. Recent studies have shown that some linear epitopes are immunogenic and can induce neutralizing antibodies [15], [18]. Such antibodies against linear epitopes should be detectable in serologic diagnostic tests. In this study, rabbit as well as chicken sera were differentiable in an indirect ELISA employing H4, H5 and H12 HA-specific peptide antigens.

For practical purposes, it should be possible to examine sera from different host species in a blocking ELISA, where antibodies in positive test sera block antibody binding sites for the antibodies present in the indicator serum. In this study, however, it was not possible to set up a blocking ELISA based on the subtype-specific HA peptide antigens and the rabbit sera as source for indicator antibodies. Even when the rabbit sera were diluted up to 1∶6400, and a chicken serum known to contain high titers of H5 antibodies was used in a high concentration (i.e. diluted 1∶5), a subtype-specific differentiation of the chicken antibodies was not possible (data not shown). This might be due to a high avidity of the antibodies presented in the H5 rabbit serum leading to a displacement of chicken antibodies. Eventually, this problem could be solved by the use of monoclonal antibodies reactive to the used peptide, instead of the polyclonal sera.

Our data provide evidence that IV HA proteins harbour more linear epitopes than found until now, and that some of these epitopes are recognized as subtype-specific by antibodies in hyperimmune animal sera. In addition, to our knowledge, this is the first study showing that AIV subtype-specific antibody differentiation is possible in an indirect ELISA assay using polyclonal sera and synthetic linear peptides representing subtype-specific epitopes. Based on these findings, further validation studies are required based on more than 1 serum sample from different host species. The most challenging part of the validation of such an antibody subtype differentiation ELISA is, to determine if the number of subtype-specific linear epitopes per subtype is reduced by the inclusion of additional subtypes in the peptide scanning analysis, and if the differential recognition of subtype-specific epitopes, in terms of ELISA sensitivity and specificity, is compromised by the use of other heterologous sera raised against genetically more closely related IV subtypes or sera from multiple IV-infected hosts. It might be necessary to use a different set of subtype-specific peptide antigens or a combination of several antigens in a final ELISA protocol. Therefore, more efforts should be undertaken to investigate the role of linear epitopes on IV surface proteins and their classification in subtype-specific and inter subtype-conserved epitopes in order to improve AIV diagnostic and vaccination concepts. Such studies involving the majority of the remaining HA subtypes are currently under way.

## Materials and Methods

### Ethics Statement

Animal experiments have been conducted according to the national and institution's guideline (SR 455.1, Tierschutzverordnung (TSchV) 23. April, 2008) of the Swiss government.

### Isolation of viral RNA, cloning of recombinant HA and generation of recombinant baculoviruses

HPAIV A/tufted duck/Switzerland/V504/06(H5N1) originated from a dead tufted duck found during the H5N1 HPAIV epidemic in 2006 in Switzerland [30]. LPAIV A/mallard/Switzerland/WV4060166/2006(H12N2) was isolated from a healthy mallard in Switzerland (Baumer, submitted). The virus isolate A/duck/Cz/56(H4N6) (LP) was kindly provided by R. Hoop, Swiss National Reference Laboratory for Avian and Rabbit Diseases, Vetsuisse Faculty, Zurich. Total RNA was extracted from these isolates with Trizol®, hybridised with primer SZA^+^ [31] at 65°C for 10 min and reverse transcribed into cDNA with SuperScript™ III Reverse Transcriptase (Invitrogen) for 10 min at room temperature followed by 1h at 50°C.

The full-length ORF of the HA genes was amplified using primer SZAHA^+^ and SZAHA^-^ [32] and Platinum® *Taq* DNA polymerase (Invitrogen) in a touch-down PCR [30]. Amplified HA cDNA was agarose gel purified and subsequently cloned into the vector plasmid pCR® 4-TOPO® (Invitrogen) and sequenced. Full-length HA ORF sequences were either newly deposited or already available on GenBank under the accession numbers AB295611 (H4), EF547197 (H5), and GQ415321 (H12). The HA ORF were reamplified with Pfu Turbo DNA Polymerase (Sigma) in fusion with a C-terminal 6xHis tag and excluding the transmembrane domain (H5-ΔTMD and H12-ΔTMD) as well as the authentic secretion signal sequence (H4-ECD) with primers AI_H5_HA_BamH1fw (atggatccgatggaaaaaatagtgcttcttc) and AI_H5_ECDHind3rev (ataagcttaatggtgatggt gatggtgttggtaagttcctattgattcc), H4_ECD_BamH1fwd (agatccgcaaaactacacaggaaaccctg) and AI_H4_ECD_PstIrev (atctgcagttaatggtgatggtgatggtggtccttatatccctgggtcaatt), or AI_H12_HA_BamH1fwd (atcggatccgatggagaagttcattgtactgag) and AI_H12_HA_SpeIrev (atcactagttaatggtgatggtgatggtgtttgtatgtagaattctcttcaag), respectively. Truncated HA genes were subsequently cloned in pCR® 4-TOPO® (Invitrogen), resequenced and transferred by DNA restriction with enzymes cutting at the primer-encoded restriction sites and ligation into the corresponding restriction sites in the baculovirus expression vector pFastBac1 (Invitrogen). For the N-terminal fusion of the truncated HA ORF to the signal sequence of the honeybee melittin (HBM) in order to enhance secretion of the recombinant HA protein [33], the expression vector pFastBacHBM was designed. A DNA cassette encoding the polypeptide MKFLVNVALVFMVVYISYIYA was cloned into the pFastBac1 vector at the authentic translation initiation site of the polyhedrin gene. To this end a PCR fragment was generated with primers FBacMBacU (gttggctacgtatactccggaatattaatagatcatggagataattaaaatgataacca) and BacHBML (ctcggatccgcatagatgtaagaaat) using plasmid pMelBacB (Invitrogen) as template. This DNA fragment was digested with the restriction endonucleases SnaBI and BamHI and ligated into the corresponding sites of pFastBac1. In this vector, the gene of interest must be inserted in frame with the HBM signal sequence at any restriction site upstream of the stop codon-containing SpeI site in the multiple cloning site. The insertion was verified by nucleotide sequencing. Generation and amplification of recombinant baculoviruses was performed with the Bac-to-Bac-System (Invitrogen) according to the manufacturer's protocol.

### Expression and purification of recombinant AIV HA

For expression of recombinant HA, suspension cultures with 20 million High Five cells (Invitrogen) were infected with recombinant baculovirus at an m.o.i. of 10 pfu/cell for 2 h at RT, shaking. Cells were then incubated at 27°C on a shaker (150 rpm) in ESF 921 (Expression Systems, Woodland, CA, U.S.A.) or Express Five serum free medium (Gibco). After 96 h cells were separated from the cell culture supernatant (ccs) by centrifugation (10 min, 1000×g, 4°C), resuspended in carbonate buffer (100 mM carbonate, 500 mM NaCl, 10 mM imidazole, pH 9.6, with proteolysis inhibitor P8849 (Sigma) 1 µl/10^6^ cells) and sonicated 3×10 sec on ice. Sonicated cells were centrifuged for 15 min at 14000 rpm and 4°C and resuspended in carbonate buffer. Aliquots of the ccs, the supernatant after sonication and the resuspended sonicated cells were used for SDS-PAGE and Western blot analysis to determine expression efficiency and solubility of the HA proteins. Positive ccs were buffer-exchanged into carbonate buffer prior to purification using a stirred ultrafiltration cell (Amicon) or by dialysis. Recombinant HA were purified with HisTrap HP 1 ml Ni-NTA-columns and the AEKTA FPLC system (Amersham Biosciences). Columns were equilibrated with carbonate buffer and loaded with the buffer-exchanged recombinant protein preparations at a flow rate of 1 ml/min, washed with 20 ml carbonate buffer containing 10 mM imidazole (1 ml/min) and eluted with an imidazole gradient from 10 mM to 500 mM in 20 ml carbonate buffer (1 ml/min). 1 ml eluate fractions were collected and analyzed by Western blot. Fractions containing the affinity-purified HA were pooled and concentrated with Centricon® YM-10 filters (Amicon).

### SDS-PAGE and Western Blot

SDS-PAGE was performed with the Mini Protean Electrophoresis system (Bio-Rad) and analyzed with the Odyssey Infrared Imaging System (LI-COR Biosciences). For Western blot, proteins were blotted after SDS-PAGE onto nitrocellulose membranes (Porablot NCL, Macherey-Nagel) using a Trans Blot SD transfer cell (Bio-Rad). After blocking with blocking buffer (LI-COR Biosciences), the recombinant HA were detected with an anti 6xHIS tag monoclonal antibody (Roche) diluted 1∶400 in blocking buffer and a polyclonal goat anti-mouse IRDye® 800CW conjugate (LI-COR Biosciences) diluted 1∶10.000 in blocking buffer and the Odyssey® Infrared Imaging System (LI-COR® Biosciences).

### Immunization of rabbits

Before immunization, the concentrated HA fractions were dialyzed in PBS (8.1 mM Na_2_HPO_4_, 1.4 mM KH_2_PO_4_, 137 mM NaCl, 2.7 mM KCl, pH 7.3) with a Slide_A_Lyzer Dialysis Cassette, 3.500 MWCO, 0.1–0.5 ml capacity (Pierce). 200–500 µg of antigen were used for each of 3 consecutive immunizations of female New Zealand White Rabbits, 1.8 kg (Charles River Laboratories) every 4 weeks.

### Epitope mapping

PepSpots® membranes for the mapping of linear and conformational epitopes were obtained from jpt Peptide Technologies GmbH, Germany. The mapping of linear epitopes was performed following the manufacturer's protocol using rabbit sera, diluted 1∶1000 and goat anti rabbit IRDye® 800CW conjugate. The signals on the PepSpots® membranes were quantified with the Odyssey Infrared Imaging System (LI-COR Biosciences).

Epitopes were identified in a homologous and a heterologous way. In the homologous system serum against H5 HA was tested on membranes displaying peptides derived from H5 HA, serum against H4 HA on membranes with peptides derived from H4 HA and serum against H12 HA on membranes with peptides derived from H12 HA. In the heterologous system polyclonal serum raised against 1 of the 3 HA antigens was tested on membranes displaying the 2 other antigens (e.g. serum against H5 was tested on membranes displaying H4 and H12 HA).

### ELISA

Peptide ELISA was performed using Reacti-Bind™ Streptavidin High Binding Capacity Coated 96-Well Plates (Pierce) and biotin-labeled synthetic subtype-specific peptides as antigens. Plates were washed with TBS washing buffer (25 mM Tris, 150 mM NaCl; pH 7.2, 0.1% BSA, 0.05% Tween-20) and coated over night at 4°C with peptides diluted in washing buffer. After coating, plates were washed and incubated either with the rabbit antisera or with an anti-H5N9 chicken serum, generated by intramuscular vaccination of chicken with a commercial inactivated H5N9 poultry vaccine and kindly provided by our in-house vaccine registration department, diluted in washing buffer for 1 h at 37°C. For indirect ELISA, sera were removed and plates were washed in washing buffer prior to incubation for 1 h 37°C with species-specific HRP-conjugates (polyclonal swine anti-rabbit HRP, Dako Cytomation) diluted 1∶4000 in washing buffer; or undiluted anti-chicken/turkey hen-IgY-HRP (Labor Diagnostik Leipzig, Germany). After removal of the conjugate and final washing, plates were incubated at room temperature with ABTS substrate containing 1% of 1% H_2_O_2_. OD values were recorded after 15, 30, 45, 60, 90 and 120 minutes at 405 nm. For blocking ELISA, plates were incubated first with test serum and then with rabbit serum without to remove the test serum, 1 h 37°C each. Conjugates and substrate were added and measurements were performed as described for indirect ELISA.

## Supporting Information

Table S1 Epitope-displaying peptides. Subtype-specific epitope sequences are underlined. Reactivities were quantified and categorised into 100% to 70% (++) and 70% to 30% (+). Peptides containing glycosylation sites are indicated with underlined numbers.
(0.04 MB XLS)

## References

[1] Dugan VG, Chen R, Spiro DJ, Sengamalay N, Zaborsky J (2008). The evolutionary genetics and emergence of avian influenza viruses in wild birds.. PLoS Pathog.

[2] Suzuki Y (2005). Sialobiology of influenza: molecular mechanism of host range variation of influenza viruses.. Biol Pharm Bull.

[3] Kalthoff D, Globig A, Beer M (2009). (Highly pathogenic) avian influenza as a zoonotic agent.. Vet Microbiol.

[4] Kelly TR, Hawkins MG, Sandrock CE, Boyce WM (2008). A review of highly pathogenic avian influenza in birds, with an emphasis on Asian H5N1 and recommendations for prevention and control.. J Avian Med Surg.

[5] OIE (2008). Manual of Diagnostic Tests and Vaccines for Terrestrial Animals.. ISBN.

[6] Prabakaran M, Ho HT, Prabhu N, Velumani S, Szyporta M (2009). Development of epitope-blocking ELISA for universal detection of antibodies to human H5N1 influenza viruses.. PLoS ONE.

[7] Starick E, Werner O, Schirrmeier H, Kollner B, Riebe R (2006). Establishment of a competitive ELISA (cELISA) system for the detection of influenza A virus nucleoprotein antibodies and its application to field sera from different species.. J Vet Med B Infect Dis Vet Public Health.

[8] Velumani S, Du Q, Fenner BJ, Prabakaran M, Wee LC (2007). Development of an antigen-capture ELISA for detection of H7 subtype avian influenza from experimentally infected chickens.. J Virol Methods.

[9] Watson DS, Reddy SM, Brahmakshatriya V, Lupiani B (2009). A multiplexed immunoassay for detection of antibodies against avian influenza virus.. J Immunol Methods.

[10] Nayak DP, Davis AR, McQueen NL, Bos TJ, Jabbar MA (1985). Biological and immunological properties of haemagglutinin and neuraminidase expressed from cloned cDNAs in prokaryotic and eukaryotic cells.. Vaccine.

[11] Schulze-Gahmen U, Klenk HD, Beyreuther K (1986). Immunogenicity of loop-structured short synthetic peptides mimicking the antigenic site A of influenza virus hemagglutinin.. Eur J Biochem.

[12] Nestorowicz A, Tregear GW, Southwell CN, Martyn J, Murray JM (1985). Antibodies elicited by influenza virus hemagglutinin fail to bind to synthetic peptides representing putative antigenic sites.. Mol Immunol.

[13] Caton AJ, Brownlee GG, Yewdell JW, Gerhard W (1982). The antigenic structure of the influenza virus A/PR/8/34 hemagglutinin (H1 subtype).. Cell.

[14] Green N, Alexander H, Olson A, Alexander S, Shinnick TM (1982). Immunogenic structure of the influenza virus hemagglutinin.. Cell.

[15] Shen S, Mahadevappa G, Oh HL, Wee BY, Choi YW (2008). Comparing the antibody responses against recombinant hemagglutinin proteins of avian influenza A (H5N1) virus expressed in insect cells and bacteria.. J Med Virol.

[16] Khurana S, Suguitan AL, Rivera Y, Simmons CP, Lanzavecchia A (2009). Antigenic fingerprinting of H5N1 avian influenza using convalescent sera and monoclonal antibodies reveals potential vaccine and diagnostic targets.. PLoS Med.

[17] Huang H, Dan H, Zhou Y, Yu Z, Fan H (2007). Different neutralization efficiency of neutralizing monoclonal antibodies against avian influenza H5N1 virus to virus strains from different hosts.. Mol Immunol.

[18] Ekiert DC, Bhabha G, Elsliger MA, Friesen RH, Jongeneelen M (2009). Antibody Recognition of a Highly Conserved Influenza Virus Epitope.. Science.

[19] Gerdon AE, Wright DW, Cliffel DE (2005). Hemagglutinin linear epitope presentation on monolayer-protected clusters elicits strong antibody binding.. Biomacromolecules.

[20] Kaverin NV, Rudneva IA, Govorkova EA, Timofeeva TA, Shilov AA (2007). Epitope Mapping of the Hemagglutinin Molecule of a Highly Pathogenic H5N1 Influenza Virus by Using Monoclonal Antibodies.. J Virol.

[21] Smirnov YA, Lipatov AS, Gitelman AK, Okuno Y, Van BR (1999). An epitope shared by the hemagglutinins of H1, H2, H5, and H6 subtypes of influenza A virus.. Acta Virol.

[22] Okuno Y, Isegawa Y, Sasao F, Ueda S (1993). A common neutralizing epitope conserved between the hemagglutinins of influenza A virus H1 and H2 strains.. J Virol.

[23] Vareckova E, Cox N, Klimov A (2002). Evaluation of the subtype specificity of monoclonal antibodies raised against H1 and H3 subtypes of human influenza A virus hemagglutinins.. J Clin Microbiol.

[24] Vareckova E, Mucha V, Kostolansky F, Gubareva LV, Klimov A (2007). HA2-specific monoclonal antibodies as tools for differential recognition of influenza A virus antigenic subtypes.. Virus Res.

[25] Zander H, Reineke U, Schneider-Mergener J, Skerra A (2007). Epitope mapping of the neuronal growth inhibitor Nogo-A for the Nogo receptor and the cognate monoclonal antibody IN-1 by means of the SPOT technique.. J Mol Recognit.

[26] Subbarao K, Murphy BR, Fauci AS (2006). Development of effective vaccines against pandemic influenza.. Immunity.

[27] Rao SS, Styles D, Kong W, Andrews C, Gorres JP (2009). A gene-based avian influenza vaccine in poultry.. Poult Sci.

[28] Jarvis DL (2003). Developing baculovirus-insect cell expression systems for humanized recombinant glycoprotein production.. Virology.

[29] Batista FD, Harwood NE (2009). The who, how and where of antigen presentation to B cells.. Nat Rev Immunol.

[30] Hofmann MA, Renzullo S, Baumer A (2008). Phylogenetic characterization of H5N1 highly pathogenic avian influenza viruses isolated in Switzerland in 2006.. Virus Genes.

[31] Hoffmann E, Stech J, Guan Y, Webster RG, Perez DR (2001). Universal primer set for the full-length amplification of all influenza A viruses.. Arch Virol.

[32] Zou S (1997). A practical approach to genetic screening for influenza virus variants.. J Clin Microbiol.

[33] Tessier DC, Thomas DY, Khouri HE, Laliberte F, Vernet T (1991). Enhanced secretion from insect cells of a foreign protein fused to the honeybee melittin signal peptide.. Gene.

